# Zoledronic acid inhibits vasculogenic mimicry in murine osteosarcoma cell line in vitro

**DOI:** 10.1186/1471-2474-12-146

**Published:** 2011-06-30

**Authors:** Dehao Fu, Xianfeng He, Shuhua Yang, Weihua Xu, Tao Lin, Xiaobo Feng

**Affiliations:** 1 Department of Orthopaedics, Union Hospital, Tongji Medical College, Huazhong University of Science and Technology, Wuhan, 430022, China; 2 Department of Orthopaedics, Ningbo No.6 Hospital, Ningbo, 315040, China

## Abstract

**Background:**

To study the effects of zoledronic acid (ZA) on the vasculogenic mimicry of osteosarcoma cells *in vitro*.

**Methods:**

A Three-dimensional culture of LM8 osteosarcoma cells on a type I collagen matrix was used to investigate whether osteosarcoma cells can develop vasculogenic mimicry, and to determine the effects of ZA on this process. In addition, the cellular ultrastructural changes were observed using scanning electron microscopy and laser confocal microscopy. The effects of ZA on the translocation of RhoA protein from the cytosol to the membrane in LM8 cells were measured via immunoblotting.

**Results:**

ZA inhibited the development of vasculogenic mimicry by the LM8 osteosarcoma cells, decreased microvilli formation on the cell surface, and disrupted the F-actin cytoskeleton. ZA prevented translocation of RhoA protein from the cytosol to the membrane in LM8 cells.

**Conclusions:**

ZA can impair RhoA membrane localization in LM8 cells, causing obvious changes in the ultrastructure of osteosarcoma cells and induce cell apoptosis, which may be one of the underlying mechanisms by which the agent inhibits the development of vasculogenic mimicry by the LM8 cells.

## Background

Solid malignant tumors require a robust blood supply to support growth and metastasis. For many years, tumor angiogenesis was regarded as the only means by which a tumor could acquire an adequate blood supply. In 1999, Maniotis *et al. *discovered a new type of blood vessel in human malignant uveal melanoma [1]. The blood vessel walls were created by deformed malignant melanoma cells and stromal cells. Because their structure was similar to regular blood vessels, they were considered to have vasculogenic mimicry (VM). Subsequent research found that tumors that harbor VM, such as malignant melanoma, synovial sarcoma, mesothelial sarcoma, and ovarian cancer, possess the following characteristics: a high degree of malignancy, uncommitted differentiation or bi-directional differentiation status, rapid growth, and a high rate of metastasis [2-5].

Osteosarcoma is the most common malignant bone cancer in adolescents and children. It usually has a high degree of malignancy, a rapid growth rate, and a tendency to metastasize. These characteristics resemble many of the tumors known to develop VM. Zoledronic acid (ZA) is a representative biphosphate, a class of compounds which are known to be potent anti-absorptives. Bisphosphonates have been widely used to treat hypercalcemia and ostealgia due to osteoporosis or osteitis deformans, and to treat malignant tumors that have metastasized to the bone [6]. Aside from the potent anti-absorptive effects of the compounds, biphosphates have also recently been recognized as anti-tumor agents [7-9]. However, the effects of ZA on the development of vasculogenic mimicry in osteosarcoma have not been reported. In the present study, we used a three-dimensional culture of LM8 osteosarcoma cells grown on a type I collagen matrix to investigate whether these cells develop vasculogenic mimicry, and to determine the effects of ZA on this process.

## Methods

### 1. Materials

ZA was obtained from Novartis (Basel, Switzerland) and clodronic acid from Berlex Laboratories(New Jersey, USA). The murine osteosarcoma cell line (LM8) was obtained from the Center of Experimental Animals at the Fourth Military Medical University(Xian, China). The RPMI 1640 medium was purchased from Hyclone (Thermo Scientific, Logan, Utah, USA) and fetal bovine serum from Minhai Biotechnology Company (Lanzhou, China). The CCK-8 kit was obtained from Dojindo Laboratories (Kumamoto, Japan) and collagenase from Wako Pure Chemical Industries (Osaka, Japan). Mouse tail type I collagen was obtained from Shengyou Biotechnology Inc. (Hangzhou, China) and the Annexin V FITC kit from Jingmei Biotechnology Inc. (Shenzhen, China). FOH and GGOH were purchased from Sigma (Saint Quentin Fallavier, France), and the inhibitor of RhoA from Calbiochem (San Diego, CA, USA). FITC-conjugated phalloidin was obtained from Sigma (St. Louis, MO, USA). All other reagents were the purest grade available.

### 2. Cell culture

Animal care and surgery were approved by the technical scientific and ethical committees of Union Hospital, Tongji Medical College, Huazhong University of Science and Technology and were performed under national and European regulations (Law by Decree n. 116/92). The LM8 osteosarcoma cells were maintained in an RPMI 1640 medium supplemented with 10% fetal bovine serum at 37°C under 5% CO_2_. Cells were subcultured when they reached 70-80% confluence by 0.25% trypsin digestion. When the newly plated cells reached the exponential growth phase, they were used for experiments.

### 3. Examination of cell proliferation using the CCK-8 assay

The LM8 cells in the exponential growth phase were plated in 96-well plates at a density of 5 × 10^4 ^cells/mL in the RPMI 1640 medium, which was then replaced with a fresh basal medium containing different concentrations of ZA (1, 5, and 10 μmol/L) 24 hours after plating. Cell proliferation was analyzed following the manufacturer's protocol. Briefly, after 24 hours treatment, 10 μL of the CCK-8 solution was added to each well, and then the cells were cultured for another 4 hours. At the termination of the experiment, the plate was read in a spectrometer at 450 nm to determine the absorbance of each well ("A value").

### 4. Preparation of type I collagen and 3-D cell culture

Type I collagen isolated from mouse tails was dissolved in 0.013 mol/L HCl to achieve a concentration of 5 mg/mL. The type I collagen solution (200 L) was mixed with 674 μL H_2_O in an eppendorf tube on ice. The mixture was added to 26 μL NaOH (0.1 mol/L) and mixed well. The new mixture was added to 100 μL of the 10 × RPMI 1640 medium and mixed well. In a 12-well plate, cover slides were placed in each well. Fifty μL of the medium mixture was added onto each slide and incubated at 37°C for one hour until the type I collagen gel solidified. LM8 cells were plated at a density of 5 × 10^5 ^cells/mL on the gel plaques. After the cells attached, a basal medium with or without ZA was added. The growth of LM8 cells on type I collagen plaques was monitored under an inverted contrast microscope. Twenty-four hours later, LM8 cells were harvested from 3D gels, by incubation at room temperature in collagenase at 10 mg/ml for 5 min with shaking, for proliferation assay.

### 5. Observation by scanning electron microscopy

LM8 cells in control and treatment groups were cultured on top of collagen gel plaques for 24 hours, and then fixed by 2.5% glutaraldehyde for 2-4 hours. They were subsequently washed with PBS 3 times and fixed with 1% osmium tetroxide for 1-1.5 hours. The cells were then washed with PBS for 30 minutes. After dehydration with an alcohol gradient, the samples were immobilized by a conductive adhesive and sprayed with carbon and gold. The samples were observed under a scanning electron microscope and images were taken. An MIAS-200 image analyzer was used to measure the maximum diameter of the cells, the number and length of microvilli, and the number and length of pseudopodia. The values are shown as the means ± standard deviation from three independent experiments.

### 6. Morphology and quantification of cell apoptosis

To assess cellular apoptosis, LM8 cells were plated on 6-well plates at a density of 5 × 10^5 ^cells/well. After cells attached, they were exposed to 5 μmol/L ZA (or the vehicle control) for 24 hours. The cover slides were removed and stained as instructed by the manufacturer of the TUNEL assay kit. We also assessed apoptosis using Annexin V/PI staining. For this study, the LM8 cells were treated with 0, 1, or 5 μmol/L ZA for 24 hours. The cells were then trypsinized and pelleted by centrifugation. Cell pellets were washed with phosphate buffer twice and resuspended in 250 μL binding buffer at a density of 1 × 10^6 ^cells/mL. One-hundred μL of the cell suspension was analyzed by flow cytometry after cells were stained with 5 μL Annexin V-FITC and 10 μL PI solution (20 μg/mL) to detect apoptosis.

### 7. Observation of the cell cytoskeleton by laser confocal microscopy

LM8 cells treated with ZA for 24 hours were washed with PBS twice and then fixed with 4% formaldehyde at 4°C for 10 minutes. The cells were then treated with 0.2% Triton X-100 at room temperature for 10 minutes. BSA (0.5%) was added to the cells for 30 minutes to non-specifically block the binding sites; then cells were washed twice with PBS. FITC-conjugated phalloidin (10 μg/mL) was added to stain the cells for 40 minutes at 37°C. The cells were then washed with PBS twice and with distilled water once, and slides were mounted using 50% glycerol. Cell morphology was examined under a laser confocal microscope.

### 8. RhoA Translocation Assay

To determine the membrane-bound (GTP-ase active form) of RhoA, LM8 cells were serum starved for 16 hours and then exposed to ZA or clodronic acid for 24 h. To obtain membrane and cytosolic fractions, the cells were washed and homogenized in a buffer containing 50 mM Tris-HCl, pH 7.4, 1 mM ethylene glycol tetraacetic acid, 1 mM ethylene diamine tetraacetic acid, 10 μg/mL leupeptin, 10 μg/mL aprotinin, 5 mM benzamidine HCl, 10 μg/mL soybean trypsin inhibitor, and 1 mM phenylmethylsulfonyl fluoride by ultrasonic polytron. The cell extract was centrifuged at 800 g for 5 minutes. The supernatant was then collected and further centrifuged at 15,000 g for 15 minutes at 4°C. The supernatant was centrifuged again at 100,000 g for 1 hour at 4°C, and then the supernatant was collected as the cytosolic fraction, and the pellet, as the membrane fraction, was then resuspended. Equal amounts (10 *μ*g) of protein from each fraction were used to perform western blot analysis for RhoA.

### 9. Statistical analyses

The SPSS11.5 statistical software package was used to analyze the data. To compare the differences between the control and treatment groups, a Student's t-test was used. For the comparison of multiple groups, ANOVA was used. Differences with p values less than 0.05 were regarded as statistically significant.

## Results

### 1. 5 μmol/L of ZA does not affect LM8 cell proliferation

The results of the CCK-8 assay showed that the absorbance (A value) of LM8 cells treated with the vehicle control or 1, 5, or 10 μmol/L ZA for 1 hour was 0.899 ± 0.020, 0.875 ± 0.008, 0.864 ± 0.016, and 0.829 ± 0.017, respectively. The data were derived from three independent experiments. There were no significant differences between the A values of the control group and the groups exposed to treatment with ZA at 1 or 5 μmol/L. However, 10 μmol/L ZA did significantly decrease the proliferation of the cells. Therefore, 5 μmol/L of ZA was used for the subsequent 3-D culture experiments.

### 2. ZA prevents the development of vasculogenic mimicry by osteosarcoma cells in vitro

**Figure 1:**
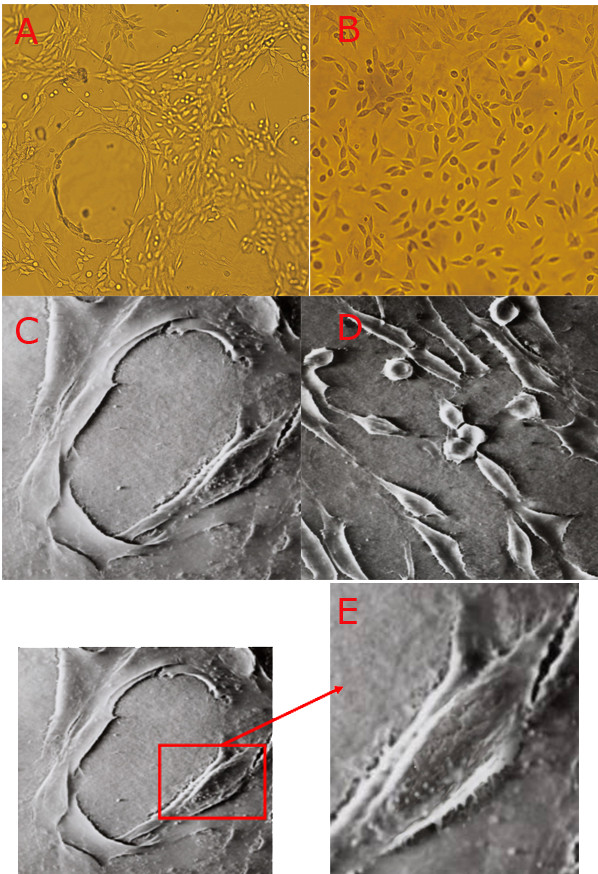
**The effect of ZA on the development of vasculogenic mimicry by LM8 osteosarcoma cells *in vitro***. The LM8 osteosarcoma cells were seeded on type I collagen gel plaques. They were treated with the vehicle control or 5 μmol/L ZA for 24 hours. The cellular structure was observed under an inverted contrast microscope (A and B, X100) or with a scanning electron microscope (C and D, X2000). E: Zoomed-in area of C.

Under inverted contrast microscopy, LM8 cells were polygonal with a full cytoplasm. Cells grown in a 3-D type-I collagen matrix culture after 24 hours partially formed long spindle shapes. Cell protrusions were obvious, and the cells formed connective structures resembling blood vessels. Exposure to 5 μmol/L ZA completely inhibited the formation of the blood vessel-like structures (Figures 1A and 1B). CCK-8 assay showed no significant differences between the A values of the control group(0.882 ± 0.012) and the 5 μmol/L ZA group(0.869 ± 0.017) (p < 0.05). The data were derived from three independent experiments.

### 3. The effects of ZA on the cellular structure of osteosarcoma cells

**Figure 2:**
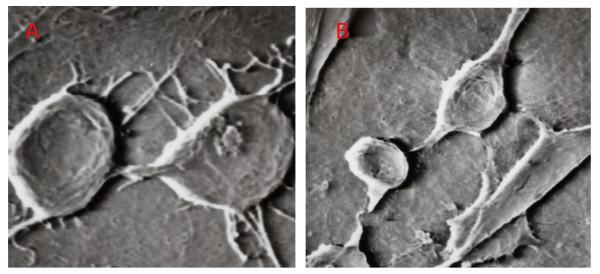
**The effect of ZA on the ultrastructures of LM8 cells grown on type I collagen gel**. The LM8 osteosarcoma cells were seeded on type I collagen gel plaques and treated with the vehicle control (A) or 5 μmol/L ZA (B) for 24 hours. The cellular ultrastructures were observed with a scanning electron microscope (X4000).

Scanning electron microscopy revealed that osteosarcoma cells formed blood vessel-like structures, and this formation was blocked by ZA treatment, confirming the observations obtained under contrast microscopy (Figures 1C and 1D). In addition, scanning electron microscopy also showed the presence of microvilli structures in the lumen of the blood vessel-like tubes formed by the osteosarcoma cells (Figure 1E). There were more microvilli on the membrane of LM8 cells in the control group compared with the ZA treatment group. The microvilli intertwined with each other, and some cells had filopodia with splitting ends. The size of the LM8 cells decreased after ZA treatment, and both the number and length of filopodia and microvilli decreased following ZA treatment (p < 0.05, Figure 2 and Table 1).

**Table 1:** Quantification of the cellular structure of LM8 osteosarcoma cells

	Control	5 μmol/L ZA	P value
Maximum cell diameter (μm)	450.46 ± 60.29	328.28 ± 48.76	P = 0.021
Number of microvilli	24.86 ± 2.68	6.48 ± 1.16	P = 0.000
Length of microvilli (μm)	87.21 ± 23.41	52.47 ± 13.18	P = 0.013
Number of filopodia	11.93 ± 0.76	4.12 ± 0.45	P = 0.000
Length of filopodia (μm)	112.56 ± 32.34	76.96 ± 14.78	P = 0.018

The values are shown as the means ± standard deviation from three independent experiments.

### 4. ZA induces apoptosis in LM8 cells

**Figure 3:**
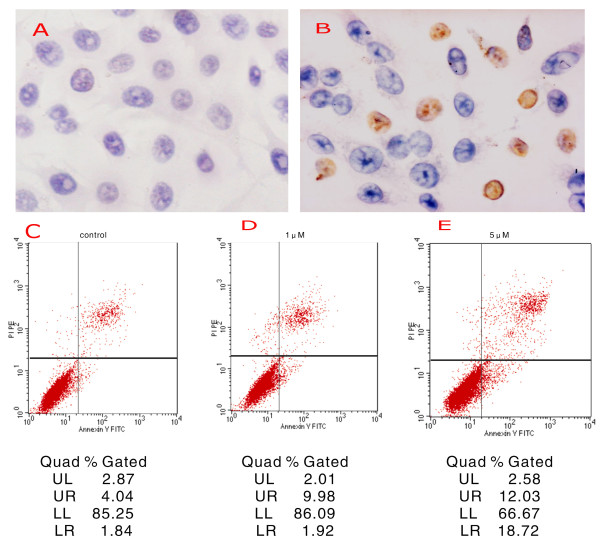
**The effect of ZA on LM8 cell apoptosis**. The LM8 osteosarcoma cells were cultured on type I collagen gel and treated with the vehicle control (A) or 5 μmol/L ZA (B) for 24 hours (X400). Apoptotic cells were stained using the TUNEL assay (brown). The non-apoptotic cells were stained blue. Alternatively, the LM8 cells treated with the vehicle control (C), 1 μmol/L ZA (D), or 5 μmol/L ZA (E) for 24 hours were analyzed by flow cytometry after staining with Annexin V-FITC/PI. The table shows the mean ± SE from *n = 3 *independent experiments, where each quadrant represents the percentage of cells in early apoptosis (*Lower right*, LR), late apoptosis (*Upper right*, UR), necrosis (*Upper left*, UL), and healthy cells (*Lower left*, LL).

LM8 cells were treated with 0, 1, or 5 μmol/L ZA for 24 hours. Cellular apoptosis was analyzed by TUNEL staining. The results showed that 5 μmol/L ZA induced extensive apoptosis (brown staining demonstrating the nuclei of apoptotic cells, Figure 3), while there were only a few apoptotic cells present in the control group. Flow cytometry using Annexin V-FITC/PI staining quantified the apoptotic cell number. These results showed that the percentage of apoptotic cells in the control cells, and those treated with 1 or 5 μmol/L ZA was 1.84 ± 0.57%, 1.92 ± 1.13%, or 18.72 ± 3.14%, respectively. The difference between the 5 μmol/L ZA group and the other two groups was statistically significant (F = 364.438, p < 0.01), while there was no statistically significant difference between the control and 1 μmol/L ZA groups (Figure 3).

### 5. The effects of ZA on the cytoskeleton of LM8 osteosarcoma cells

**Figure 4:**
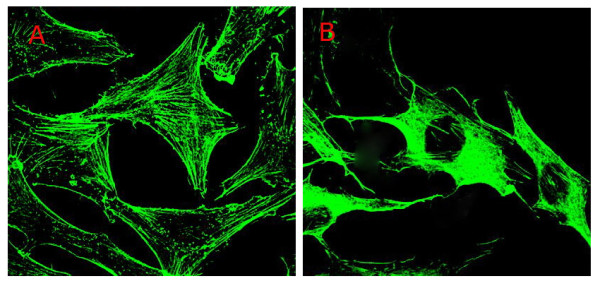
**The effect of ZA on F-actin in LM8 cells**. The LM8 osteosarcoma cells were cultured on type I collagen gel and treated with the vehicle control (A) or 5 μmol/L ZA (B) for 24 hours. F-actin was detected by green fluorescent phalloidin staining and confocal microscopy.

F-actin is one of the major components of the cytoskeleton. Phalloidin can specifically bind to F-actin. Therefore, fluorescence-conjugated phalloidin was used to detect the cytoskeleton in treated and untreated LM8 cells. Under a laser confocal microscope, F-actin was distinct in the control group, showing a compact and directional alignment with obvious fibrous tension. On the other hand, F-actin was aligned loosely in the 5 μmol/L ZA treatment group. These cells lost the fibrous and directional characteristics of the F-actin (Figure 4). Our results indicate that ZA caused the disruption of the cytoskeleton structure of LM8 cells.

### 6. ZA prevents RhoA membrane localization in LM8 cells

**Figure 5:**
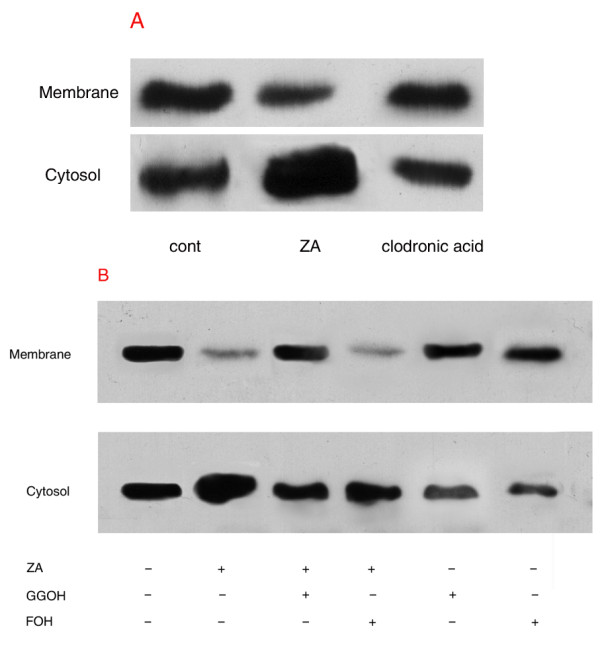
**ZA prevents RhoA membrane localization in LM8 cells**. Treatment of LM8 cells with ZA (10 μM) or clodronic acid (10 μM) for 24 hours (A). In the presence or absence of GGOH(20 μM) or FOH(20 μM) for 48 hours(A). Cells were extracted and separated intocytosolic and membrane fractions to detect RhoA by immunoblotting as described in the experimental section. These results are representative of three independent experiments.

Since the small GTPase RhoA must be targeted to the plasma membrane for its activation, we examined the effects of ZA on the translocation of RhoA protein from the cytosol to the membrane in LM8 cells after separation of cytosolic and membrane fractions. In untreated cells, equivalent amounts of RhoA are present in both fractions. Treatment of LM8 cells with ZA (10 *μ*M) for 24 h decreased membrane localization of RhoA with a reciprocal concomitant increase in RhoA in the cytosol (Figure 5A). By contrast, treatment with 10 *μ*M clodronic acid for 24 h has no the same effects. In addition, we observed that the inhibiting effect of ZA on the RhoA membrane localisation was prevented by the addition of GGOH(20 *μ*M), but not FOH(20 *μ*M) (Figure 5B).

## Discussion

In the traditional 2-D cell culture system, cells are cultured under an isolated and artificial environment that is distinct from their native physiological environment. Therefore, their biological characteristics may change dramatically. Type I collagen is the most abundant component of the extracellular matrix. It is soluble under acidic conditions. When neutralized, it can solidify to a gel form at 37°C. Type I collagen is the most widely used matrix in 3-D cell culture. Cells that grow on type I collagen gel attach to the extracellular matrix and demonstrate growth behaviors that more closely resemble their *in vivo *growth. More important, cells that grow embedded in the type I collagen gel show a similar phenotype as that of *in vivo *tumor cells. Therefore, this model has been regarded as a good platform to study the growth behaviors of tumor cells *in vitro*.

However, one weakness of the model is that the cells grow dimensionally on different plates, therefore making observations with a regular optical microscope difficult. For this reason, we have improved our 3-D culture methodology to use a thin layer of collagen to provide a 3-D growth platform, thus making optical microscopy possible. Our new methodology also distinguishes itself from traditional gel-embedded culture models by accelerating the formation of osteosarcoma vasculogenic mimicry. Twelve hours after cell plating, regular vasculogenic mimicry can be observed, whereas 3-5 days are needed if the cells are grown using a conventional gel-embedded model. In the current study, we successfully established a thin-layer 3-D culturing system using a type I collagen matrix to grow LM8 cells. Vasculogenic mimicry was developed, demonstrating that osteosarcoma cells possess metamorphic abilities and can exhibit vasculogenic mimicry *in vitro*.

Previous studies have shown that ZA can effectively inhibit the proliferation of osteosarcoma cells and induce apoptosis [1,10]. In the present study, we showed that 5 μmol/L ZA had no inhibitory effect on cell proliferation by the CCK-8 assay. Therefore, the inhibition of vasculogenic mimicry by the 5 μmol/L concentration of ZA was not due to inhibition of cell proliferation. Both optical and scanning electron microscopy showed that the 5 μmol/L concentration of ZA inhibited the development of vasculogenic mimicry by the osteosarcoma cells.

Using the TUNEL assay, we demonstrated that 5 μmol/L ZA induced apoptosis in LM8 cells. The data was validated and apoptosis was further quantified by flow cytometry using Annexin V-FITC/PI staining. Taken together, our data showed that although the 5 μmol/L concentration of ZA did not have any significant effect on cell proliferation, it promoted apoptosis.

Tumor cells possess a large number of microvilli and filopodia on the cell surface. Filopodia helps cells to move, attach, absorb nutrients, endocytose, and support cellular structures. Our current study showed that the number of microvilli and filopodia decreased dramatically following ZA treatment as observed under a scanning electron microscope. The cytoskeleton is an important component of the cellular architecture that determines the structure of the cell. The cytoskeleton connects with the plasma membrane and nuclear membrane proteins, and is important in supporting the normal cell shape. Moreover, the cytoskeleton is involved in cell mobility and division, and forms the basic structure for transmembrane signal transduction. Cells rely on the cytoskeleton to survive, divide, grow, and interact with other cells. Actin is present in cells in either the conjugated form (F-actin) or the isolated form (G-actin). G-actin is a single-unit globular protein that is soluble in the cytoplasm. In contrast, F-actin is fibrous and is the major component of the cytoskeleton. F-actin is more involved in biological processes than G-actin. Microfilaments are comprised of two strands of F-actin that have combined in a helical form. In the resting state, the two forms of actin (F- and G-actin) are in equilibrium to maintain the morphology and functions of the cell. In the current study, F-actin in LM8 osteosarcoma cells treated with ZA lost its fibrous and directional characteristics as indicated by laser confocal microscopy, resulting in disruption of the cytoskeletal structure. Overall, our results indicated that ZA can induce apoptosis in LM8 cells, decrease the number of microvilli and filopodia on the cell surface, and disrupt the structure of F-actin in the cytoskeleton. All of these effects may contribute to the inhibitory effects of ZA on the development of vasculogenic mimicry, future investigations should clarify this point.

However, our study has several limitations. First, only an in vitro approach was used in the study. We therefore cannot ensure how apoptosis and vasculogenic mimicry are connected. Thereby, to confirm the capability of LM8 to organize a vasculogenic mimicry network, an in vivo or ex-vivo model should be established in future study. Second, ZA is a nitrogen-containing bisphosphonate and could inhibit the expression of Rho GTPase geranylgeranylation, which family has been shown to regulate many aspects of intracellular actin dynamics (lamellipodia, filopodia, actin retraction, phagocytosis, cell morphology) [11], in Saos-2 osteoblast-like cells [12], MDA-MB-231 breast cancer cell line [13] and multiple myeloma RPMI8226 cell line [14]. In this study, We found that ZA impairs RhoA translocation from the cytosol to the membrane in LM8 cells, while clodronic acid (a no nitrogen-containig bisphosphonate) has no same effects. This pharmacodynamic aspect provides a potential mechanism to explain how ZA is capable to determine F-actin cytoskeleton and ultrastructural modifications (thus affecting vasculogenic mimicry development). Research on the molecular mechanisms underlying VM and the functional relevance will improve our understanding of this process and may ultimately contribute to the development of more accurate treatment modalities [15].

## Conclusions

ZA can prevent RhoA membrane localization in LM8 cells, causing obvious changes in the ultrastructure of osteosarcoma cells and induce cell apoptosis, which may be one of the underlying mechanisms by which the agent inhibits the development of vasculogenic mimicry by the LM8 cells.

## Competing interests

The authors declare that they have no financial or non-financial competing interests. Novartis has no involvement in the funding or design of the study.

## Authors' contributions

All authors read, edited, and approved the final manuscript. WX and SY are the lead investigators, and developed the design of the study, carried out data-acquisition, analysis, interpretations, and prepared as primary authors for the manuscript. DF, XH, and TL were responsible for the design, project supervision, and writing of the manuscript. XF assisted in carrying out data acquisition and was involved in preparing the study design and in writing the manuscript.

## Pre-publication history

The pre-publication history for this paper can be accessed here:

http://www.biomedcentral.com/1471-2474/12/146/prepub

## References

[B1] Maniotis AJ Folberg R Hess A Seftor EA Gardner LM Pe'er J Trent JM Meltzer PS Hendrix MJ Vascular channel formation by human melanoma cells in vivo and in vitro: vasculogenic mimicry Am J Pathol 1999 155 739 752 10.1016/S0002-9440(10)65173-5 10487832 PMC1866899

[B2] Ahmadi SA Moinfar M Gohari Moghaddam K Bahadori M Practical application of angiogenesis and vasculogenic mimicry in prostatic adenocarcinoma Arch Iran Med 2010 13 498 503 21039005

[B3] Xu X Jia R Zhou Y Song X Fan X Investigation of vasculogenic mimicry in sebaceous carcinoma of the eyelid Acta Ophthalmo 2010 88 e160 164 10.1111/j.1755-3768.2010.01942.x 20553231

[B4] El Hallani S Boisselier B Peglion F Rousseau A Colin C Idbaih A Marie Y Mokhtari K Thomas JL Eichmann A Delattre JY Maniotis AJ Sanson M A new alternative mechanism in glioblastoma vascularization: tubular vasculogenic mimicry Brain 2010 133 973 982 10.1093/brain/awq044 20375132 PMC4861203

[B5] Clemente M Pérez-Alenza MD Illera JC Peña L Histological, immunohistological, and ultrastructural description of vasculogenic mimicry in canine mammary cancer Vet Patho 2010 47 265 274 10.1177/0300985809353167 20106772

[B6] Dhillon S Lyseng-Williamson KA Zoledronic acid: a review of its use in the management of bone metastases of malignancy Drugs 2008 68 507 534 10.2165/00003495-200868040-00010 18318568

[B7] Burkinshaw R Winter M Neville-Webbe H Lester J Woodward E Brown J Zoledronic acid Expert Opin Drug Saf 2011 10 133 145 10.1517/14740338.2011.540387 21114419

[B8] Green J Lipton A Anticancer properties of zoledronic acid Cancer Invest 2010 28 944 957 10.3109/07357907.2010.512598 20879838

[B9] Ottewell PD Lefley DV Cross SS Evans CA Coleman RE Holen I Sustained inhibition of tumor growth and prolonged survival following sequential administration of doxorubicin and zoledronic acid in a breast cancer model Int J Cancer 2010 126 522 532 10.1002/ijc.24756 19621384

[B10] Xian-feng HE Shu-hua YANG De-hao FU Jia-guo LIU Yong HU Yu HE Qing-de WANG Effect of zoledronic acid on proliferation, apoptosis and expression of VEGF of osteosarcoma cell line LM8 China Oncology 2007 17 531 535

[B11] Yuasa T Kimura S Ashihara E Habuchi T Maekawa T Zoledronic acid - a multiplicity of anti-cancer action Curr Med Chem 2007 14 2126 2135 17691952 10.2174/092986707781389600

[B12] Chaplet M Detry C Deroanne C Fisher LW Castronovo V Bellahcéne A Zoledronic acid up-regulates bone sialoprotein expression in osteoblastic cells through Rho GTPase inhibition Biochem J 2004 384 591 598 10.1042/BJ20040380 15324309 PMC1134145

[B13] Denoyelle C Hong L Vannier JP Soria J Soria C New insights into the actions of bisphosphonate zoledronic acid in breast cancer cells by dual RhoA-dependent and -independent effects Br J Cancer 2003 88 1631 1640 10.1038/sj.bjc.6600925 12771933 PMC2377117

[B14] Koizumi M Nakaseko C Ohwada C Takeuchi M Ozawa S Shimizu N Cho R Nishimura M Saito Y Zoledronate has an antitumor effect and induces actin rearrangement in dexamethasone-resistant myeloma cells Eur J Haematol 2007 79 382 391 10.1111/j.1600-0609.2007.00957.x 17903213

[B15] Paulis YW Soetekouw PM Verheul HM Tjan-Heijnen VC Griffioen AW Signalling pathways in vasculogenic mimicry Biochim Biophys Acta 2010 1806 18 28 20079807 10.1016/j.bbcan.2010.01.001

